# Efficient Multivariate Time-Series Forecasting via Latency-Constrained Distillation and Dynamic Pruning

**DOI:** 10.3390/e28101104

**Published:** 2026-10-10

**Authors:** Yuting Bai, Xixian Zhang, Jianlei Kong, Xuebo Jin, Tingli Su

**Affiliations:** 1 National Engineering Research Center for Agri-Product Quality Traceability Technology and Application, Beijing Technology and Business University, Beijing 102488, China; 2 School of Computer and Artificial Intelligence, Beijing Technology and Business University, Beijing 102488, China

**Keywords:** multivariate time-series forecasting, knowledge distillation, hardware-aware optimization, dynamic structured pruning, inference latency

## Abstract

Multivariate time-series forecasting models often achieve improved prediction accuracy at the cost of increased model complexity and inference latency, limiting their deployment on resource-constrained computing platforms. In addition, lower parameter counts or theoretical computational costs do not necessarily lead to faster execution on actual hardware. To address this issue, we propose a Latency-Constrained Distillation and Dynamic Pruning (LCDP) framework for efficient multivariate time-series forecasting. LCDP introduces a latency-constrained adaptive optimization strategy that jointly considers forecasting loss, knowledge-distillation loss, local feature-alignment loss, and hardware inference latency during student-model training. Local feature-guided knowledge transfer is used to enhance the student model’s ability to capture fine-grained temporal patterns, while deployment-oriented dynamic structural pruning progressively adjusts the group-wise sparsity pattern of the student model according to latency feedback from the target hardware while retaining the original dense layer dimensions. Experiments on the ETTh1, ETTm1, and ETTm2 datasets using two CPU platforms show that LCDP achieves competitive forecasting accuracy with a lightweight student inference backbone containing 7336 active parameters and requiring 50,848 dense multiply-accumulate operations. The saved student checkpoint additionally contains 2080 parameters in a training-time feature-alignment projection that is not invoked during inference. LCDP provides an effective balance between forecasting accuracy and practical inference efficiency, demonstrating its potential for low-latency multivariate time-series forecasting on CPU-based resource-constrained platforms.

## 1. Introduction

Multivariate time-series forecasting is widely used in energy scheduling, industrial monitoring, and traffic management, where it supports system-state analysis, operational decision-making, and risk warning. In recent years, deep neural networks have improved forecasting accuracy, but complex network structures typically involve large parameter counts and high computational costs. When such models are deployed on resource-constrained computing platforms, limited computing and storage capacity may reduce inference efficiency and compromise real-time forecasting. Multivariate time-series forecasting for resource-constrained applications must therefore account not only for forecasting accuracy but also for model size, computational cost, and inference efficiency [1]. Recent studies have also demonstrated the broad applicability of deep forecasting models in traffic-flow and retail-sales prediction [2,3].

Lightweight time-series forecasting has consequently become an important research direction for reducing computational cost. Existing approaches mainly include lightweight network design, knowledge distillation, and pruning-based compression, which improve efficiency by simplifying model structures, transferring the predictive capability of complex models, or removing redundant computational units. However, these approaches generally focus on theoretical complexity measures, such as parameter count and computational cost, while paying comparatively little attention to actual inference latency on the target computing platform. Because inference is also affected by model structure, computational operators, and hardware execution characteristics, lower theoretical complexity does not necessarily translate directly into faster inference. Establishing an inference-efficiency-oriented optimization mechanism that aligns model compression with actual runtime performance therefore remains an important challenge in lightweight time-series forecasting.

In multivariate time-series forecasting, recurrent neural networks (RNNs), temporal convolutional networks (TCNs), Transformer [4], PatchTST [5], and related variants have improved the modeling of complex temporal dependencies through recurrent, convolutional, or attention-based structures. However, as model size and structural complexity increase, parameter count and computational cost also grow, limiting deployment on resource-constrained platforms. Conversely, although existing lightweight forecasting models can reduce model size and computational cost, their limited capacity may weaken their ability to represent complex temporal patterns and inter-variable relationships. Maintaining expressive temporal representations while reducing inference overhead is therefore another challenge for efficient time-series forecasting.

Overall, existing lightweight time-series forecasting methods have several limitations. First, knowledge-distillation methods mainly improve lightweight models by transferring output predictions or intermediate features, but they generally lack explicit constraints on inference latency; the resulting student models may therefore not run more efficiently. Second, most distillation strategies focus on global prediction outputs or representations and have limited ability to transfer local temporal patterns and fine-grained feature information, which can constrain lightweight models in scenarios with complex dynamics. Finally, existing pruning methods generally compress structures using fixed ratios, static thresholds, or empirical rules, without a unified closed-loop mechanism linking model training, structural optimization, and deployment-efficiency evaluation. Reducing parameter count or computational cost alone is therefore insufficient to guarantee both forecasting accuracy and inference efficiency. A coordinated optimization framework that integrates knowledge transfer, latency-constrained optimization, and dynamic structural adjustment is needed.

To address these issues, we propose an efficient multivariate time-series forecasting framework termed Latency-Constrained Distillation and Dynamic Pruning (LCDP). The framework reduces inference overhead while maintaining forecasting performance by coordinating knowledge transfer, latency-constrained optimization, and dynamic structural adjustment. Specifically, Latency-Constrained Adaptive Optimization (LCAO) incorporates prediction error, distillation error, and a target-latency constraint into a unified optimization process, allowing model training to account for both forecasting accuracy and inference efficiency. Local feature-guided knowledge transfer uses local temporal representations from the teacher model to strengthen the lightweight student model’s ability to represent complex temporal patterns. Deployment-Oriented Dynamic Structural Pruning (DOSP) progressively adjusts the group-wise sparsity pattern of the student model according to latency feedback from the target computing platform while retaining the original dense layer dimensions. Its effect is evaluated using forecasting accuracy and measured CPU latency rather than reductions in the registered checkpoint parameter count or dense inference MACs.

The main contributions of this study are summarized as follows:

(1) We propose the LCDP framework, which integrates knowledge transfer, latency-constrained optimization, and dynamic structural adjustment. Unlike conventional lightweight approaches that assess model efficiency mainly through parameter count or computational cost, LCDP jointly considers forecasting performance and inference efficiency.
(2) We design an LCAO mechanism that incorporates prediction loss, knowledge-distillation loss, local feature-alignment loss, and a latency-constraint term into the training objective. The student model can therefore learn the teacher model’s predictive capability while adapting its optimization direction to the target inference-efficiency requirement.
(3) We introduce local feature-guided knowledge transfer and deployment-oriented dynamic structural pruning. The former transfers local temporal feature representations from the teacher model to alleviate the limited representational capacity of the lightweight model, whereas the latter progressively adjusts the structured sparsity level according to latency feedback from the target computing platform, avoiding reliance on a single fixed pruning ratio.

Experiments on multiple public time-series datasets show that the proposed method reduces actual inference latency while maintaining forecasting accuracy, providing an approach to efficient time-series forecasting in resource-constrained environments.

## 2. Related Work

### 2.1. Multivariate Time-Series Forecasting Models

Traditional time-series forecasting methods mainly include statistical models such as the autoregressive integrated moving average (ARIMA) model and vector autoregression (VAR). These methods typically rely on predefined statistical assumptions and apply to linear, stationary, or low-dimensional time series. However, their modeling capacity can be limited for data with nonlinear variations, long-term dependencies, or strong multivariate interactions. Compared with conventional statistical models, deep-learning methods can learn complex temporal features through multilayer nonlinear structures and offer advantages in representing long-range dependencies and multivariate relationships. RNNs and their variants, including long short-term memory (LSTM) networks and gated recurrent units (GRUs), were among the first deep-learning models applied to time-series forecasting. TCNs extract temporal features using causal and dilated convolutions. Nevertheless, these models may still encounter limitations in training stability and representational capacity for long-sequence forecasting and high-dimensional multivariate inputs. For example, RNN-based models are susceptible to vanishing gradients when modeling long-term dependencies, whereas TCNs may require greater network capacity to maintain forecasting performance for complex high-dimensional inputs.

As long-sequence forecasting has received increasing attention, a range of Transformer-based time-series forecasting models have been proposed, including Informer [6], Autoformer [7], FEDformer [8], PatchTST, iTransformer [9], and TimesNet [10]. These models generally capture long-range dependencies through self-attention, frequency-domain modeling, patch-based modeling, or dimension inversion and can therefore be effective for long-sequence modeling. However, they often require many network parameters and computational operations, particularly when the input sequence is long or the number of variables is large. Although Transformer-based models can improve long-sequence forecasting, deployment on resource-constrained platforms still requires careful consideration of model size and inference efficiency. Recent studies have further emphasized fair benchmarking and dataset heterogeneity [11], adaptive modeling of time-varying cross-variable relations [12], and multi-period dynamics in multivariate forecasting [13].

In summary, existing multivariate time-series forecasting models primarily emphasize forecasting accuracy and the capacity to model complex temporal relationships, often increasing model size and structural complexity to improve feature representations. However, larger parameter counts and greater computational demands limit their use on resource-limited platforms. Simply reducing model size can lower computational cost but may weaken the representation of complex temporal dependencies and multivariate interactions. Achieving an effective balance between forecasting performance and inference efficiency while retaining strong temporal modeling capacity therefore remains an important problem in efficient multivariate time-series forecasting.

### 2.2. Lightweight and Efficient Forecasting Methods

Lightweight time-series forecasting has received increasing attention as a means of reducing the computational cost of forecasting models. Existing studies generally simplify either the model structure or the computational process through lightweight network design, low-rank modeling, channel-independent modeling, or simplified mappings [14,15,16]. Lightweight network design reduces parameter count by decreasing the number of layers, hidden dimensions, or modules. Low-rank modeling compresses matrix representations to reduce computation. Channel-independent modeling decomposes a multivariate series into relatively independent univariate modeling processes. Simplified mapping methods use linear layers or shallow networks to transform inputs directly into forecasts. These approaches can reduce model complexity and, to some extent, improve inference efficiency on resource-constrained platforms. More recent lightweight designs employ high-dimensional feature maps [17] and hybrid state-space architectures [18] to reduce computational overhead while retaining competitive long-horizon forecasting performance.

GRU, Transformer, and Informer are commonly used as representative baselines in multivariate time-series forecasting. A GRU captures sequential dependencies through gating mechanisms and provides a more stable training process than a standard recurrent neural network. Transformer captures long-range dependencies through self-attention, whereas Informer simplifies attention computation for long-sequence forecasting. Although these models represent established approaches to temporal feature modeling, their inference processes still involve substantial matrix computation or sequential state updates, and their runtime efficiency on resource-constrained platforms warrants further consideration.

Several lightweight architectures have been developed to reduce the parameter count and computational cost of forecasting models. The Basic Echo State Network (BESN) employs a fixed recurrent reservoir to capture nonlinear temporal dynamics and trains only the output mapping [19]. DLinear [20] decomposes a series into trend and seasonal components and simplifies complex temporal mapping into a linear forecasting process, thereby achieving low structural complexity. These methods offer advantages in inference speed and model size, but their relatively limited capacity may constrain forecasting performance when modeling complex nonlinear variations or high-dimensional interactions.

KAN [21] and Token Merging [22] provide alternative approaches to efficient time-series forecasting. KAN uses learnable function mappings to strengthen the representation of nonlinear relationships, offering a new structural option for modeling complex temporal patterns. Token Merging combines redundant tokens at the input-representation or intermediate-feature level to reduce the number of sequence units processed in subsequent computations. Rather than directly reducing the number of network layers or hidden dimensions, these approaches adjust the representation and computational path, but practical applications must still balance forecasting accuracy, computational cost, and hardware latency.

Overall, existing lightweight time-series forecasting approaches reduce model complexity through network simplification, representation compression, and computational-path optimization, thereby improving runtime efficiency to some extent. However, most approaches assess lightweight performance using parameter count, computational cost, or predefined compression rules and lack an explicit optimization mechanism for inference latency on the target computing platform. Fixed structural designs and static compression strategies are also difficult to adapt to different computing environments and runtime requirements. Moreover, reducing model capacity may weaken the representation of complex temporal patterns, creating a trade-off between forecasting accuracy and inference efficiency. Lightweight time-series forecasting therefore requires further investigation of optimization methods that jointly consider latency constraints, model representational capacity, and structural adaptability. Recent comparative evidence also suggests that increasing forecasting-model scale does not necessarily yield proportional gains over compact alternatives, reinforcing the need to evaluate predictive performance together with practical resource efficiency [23].

### 2.3. Knowledge Distillation and Pruning

Knowledge distillation is a widely used method for model compression and knowledge transfer. Its basic principle is to use a teacher model with strong modeling capacity to guide a smaller student model, allowing the student to learn the teacher’s outputs or intermediate representations at a lower computational cost [24]. Distillation has also been applied to lightweight time-series forecasting. For example, Lai et al. proposed LightCTS [25], which uses model distillation to improve a lightweight model for correlated time-series forecasting, and Ni et al. proposed TimeDistill [26], which transfers temporal and frequency-domain patterns from a complex teacher model to a lightweight multilayer perceptron. Existing time-series distillation methods mainly include output distillation, feature distillation, and multi-layer representation alignment. Output distillation encourages the student to approximate the teacher’s forecasts, whereas feature distillation and representation alignment further use intermediate information from the teacher model. Knowledge distillation can therefore alleviate the limited representational capacity of lightweight student models and provide a viable basis for efficient time-series forecasting.

Although knowledge distillation can help a lightweight student model acquire the teacher model’s predictive capability, existing distillation methods generally emphasize consistency in prediction outputs or intermediate features and give limited consideration to inference latency, hardware cost, and deployment environments. A student model may achieve strong forecasting performance through distillation without attaining low inference latency on the target hardware. In addition, distillation mainly operates during model training and does not directly constrain redundant computational paths in the model structure. Knowledge distillation alone is therefore insufficient to meet the joint requirements of forecasting accuracy and runtime efficiency in resource-constrained scenarios.

Network pruning is a common model-compression technique that removes parameters, connections, or structural units with limited contributions to reduce model size and computational cost. According to the pruning target, pruning methods can generally be divided into unstructured and structured pruning. Unstructured pruning removes individual weights or connections and can produce high sparsity, but it does not necessarily yield stable inference acceleration on general-purpose hardware. Structured pruning directly removes channels, neurons, attention heads, or network modules and is therefore easier to align with existing deep-learning frameworks and hardware execution patterns [27]. Pruning and sparsification have also recently been applied to time-series forecasting. For example, studies have compared unstructured and structured pruning in Transformer-based forecasting models and analyzed their effects on model size, computational cost, and inference time [28]. Zhao et al. proposed a structured pruning method for time-series foundation models to obtain a more compact task-specific parameter space [29]. Overall, pruning reduces computational complexity by removing redundant connections, channels, or modules, and structured pruning is generally more practical for deployment and hardware acceleration.

Knowledge distillation and network pruning provide effective routes to lightweight time-series forecasting, but both leave room for improvement. Existing knowledge-distillation methods mainly transfer teacher-model outputs or global feature representations; they make comparatively limited use of local temporal patterns and fine-grained dynamic information and generally lack joint constraints on inference efficiency. Existing pruning methods, meanwhile, typically compress model structures according to parameter importance or manually specified rules. The pruning process is often independent of model training and inference-performance evaluation, making it difficult to adapt to runtime feedback from the target platform. Distillation and pruning are also commonly treated as separate compression steps without a unified optimization mechanism. An important open problem is therefore how to strengthen the lightweight model’s representations through effective knowledge transfer while jointly optimizing forecasting performance and inference efficiency through latency constraints and dynamic structural adjustment.

### 2.4. Summary of Existing Limitations and Motivation

In summary, existing multivariate time-series forecasting models, lightweight methods, knowledge distillation, and pruning techniques provide multiple routes to greater model efficiency, but they retain several limitations for efficient inference. First, existing methods generally optimize prediction error, parameter count, or theoretical computational cost and rarely impose explicit constraints on actual inference latency on the target computing platform. Second, knowledge transfer, model compression, and structural optimization are commonly performed as relatively independent processes without a unified coordination mechanism. Finally, reducing computational cost may weaken the lightweight model’s ability to represent complex temporal features, whereas existing structural-compression methods cannot readily adjust the model structure in response to runtime feedback.

Based on this analysis, we propose the LCDP framework to coordinate forecasting accuracy, model representational capacity, and inference efficiency. The framework introduces inference latency into model training through latency-constrained adaptive optimization, strengthens the lightweight model’s representation of complex temporal patterns through local feature-guided knowledge transfer, and applies latency-guided structured sparsification according to runtime feedback from the target computing platform. LCDP thereby establishes a coordinated relationship among training optimization, knowledge transfer, and post-training structured sparsification.

## 3. Latency-Constrained Distillation and Dynamic Pruning Framework

### 3.1. Problem Formulation and Overall Framework

We propose LCDP for multivariate time-series forecasting in real-world deployment scenarios. The framework aims to reduce actual inference latency on the target hardware platform while maintaining forecasting accuracy. LCDP uses joint training of teacher and student models. The teacher provides strong temporal representations and predictive knowledge, whereas the student serves as the lightweight forecasting model for final deployment. During training, a latency constraint is introduced into the optimization objective to guide the student model toward a balance among prediction error, distillation error, and inference efficiency. A dynamic pruning mechanism then applies latency-guided group-wise structured sparsification to the trained student model while retaining the original dense layer dimensions. By combining teacher-model guidance, latency-constrained optimization, and dynamic structured sparsification, LCDP provides an integrated framework for jointly optimizing forecasting accuracy and deployment efficiency.

**Figure 1:**
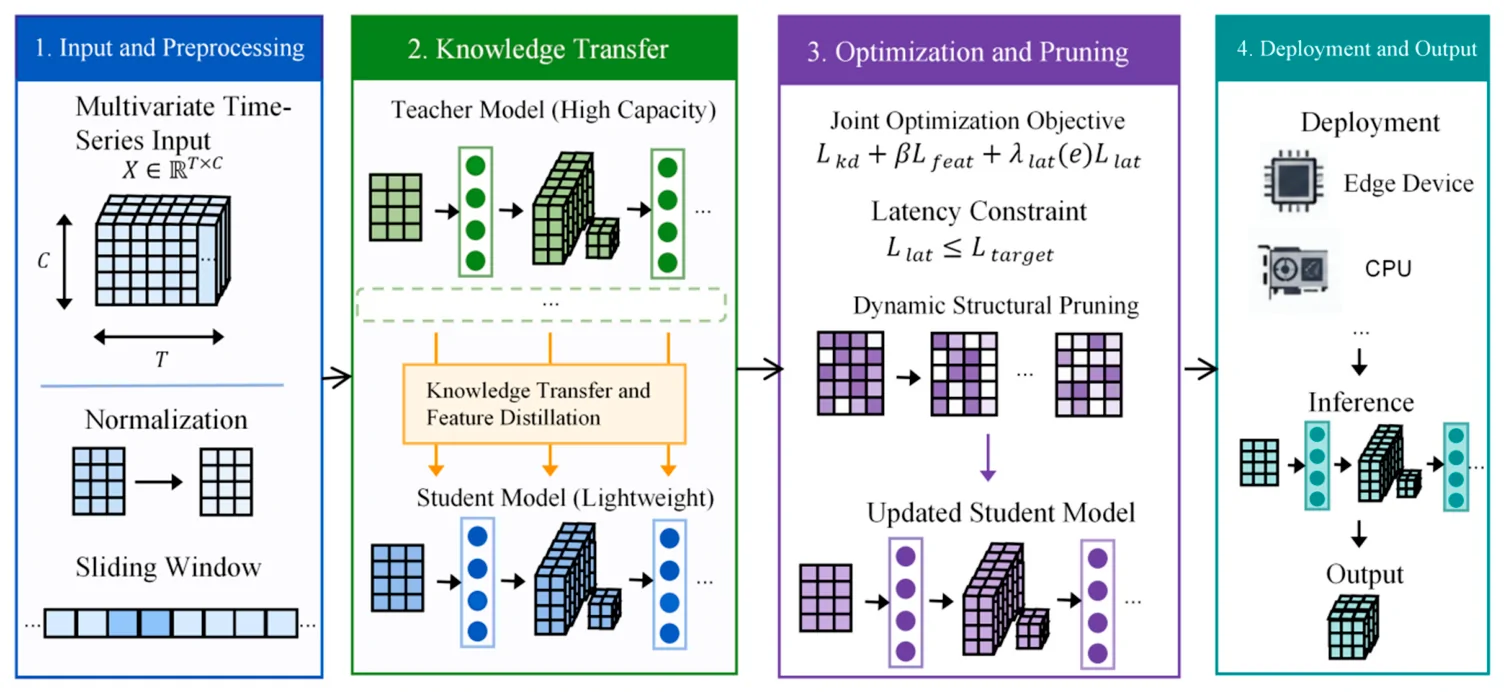
Architecture of the proposed Latency-Constrained Distillation and Dynamic Pruning framework.

As shown in Figure 1, the proposed LCDP framework consists primarily of input and preprocessing, a teacher model, a student model, local feature-guided knowledge transfer, latency-constrained adaptive optimization, and deployment-oriented dynamic structural pruning. First, multivariate time-series inputs are normalized and converted into sliding windows before being supplied to both the teacher and student models. The higher-capacity teacher model extracts richer temporal features and produces teacher forecasts and intermediate representations, whereas the lightweight student model performs feature extraction and target forecasting. The local feature-guided knowledge-transfer module then constrains both the outputs and intermediate features of the teacher and student models, allowing the student to learn local temporal patterns and representational information from the teacher. On this basis, the latency-constrained adaptive optimization module incorporates prediction error, distillation error, and a target-latency constraint into a unified optimization process, guiding the student toward a balance between forecasting accuracy and inference efficiency. After distillation training, the model enters the deployment-oriented structured-sparsification stage. Candidate pruning ratios are evaluated using latency feedback from the target hardware, after which the selected sparsified student model is briefly fine-tuned. The resulting model retains the original dense layer dimensions and is then evaluated on the target CPU platform.

The teacher model is implemented as a feed-forward multilayer perceptron consisting of a channel-independent temporal encoder and a cross-channel output projection. For each input variable, the 96-step input sequence is processed through three fully connected temporal layers with dimensions 96 → 64 → 64 → 96, with ReLU activation applied after the first two layers. The outputs of the seven input variables are then aggregated through a 7 → 1 cross-channel linear projection to generate the 96-step forecast for the target variable OT. The teacher model has a hidden dimension of 64 and contains 16,616 trainable parameters. The student model follows the same overall forecasting structure but uses a hidden dimension of 32. Its inference backbone contains 7336 parameters. During training, a 64 → 32 linear projection maps the 64-dimensional teacher representation to the 32-dimensional student feature space for local feature alignment, adding 2080 trainable parameters (64 × 32 weights and 32 biases). The saved student checkpoint therefore contains 9416 registered parameters. This feature-alignment projection is not called by the student’s forward inference path and contributes neither to the reported 50,848 dense inference MACs nor to measured inference latency. In the current implementation, the projection remains stored in the checkpoint but does not participate in deployed prediction. The teacher model is used only during training and is not included in the final inference model.

LCDP comprises a teacher model, a student model, a local feature-guided knowledge-transfer module, a latency-constrained adaptive optimization module, and a dynamic structured-sparsification module. The teacher is used only during training to provide relatively complete temporal representations and forecasting information as supervision for the lightweight student. The student is the final deployed model and uses a simpler structure for low-latency forecasting. Because the student’s small parameter count can limit its temporal representational capacity when trained directly, local feature-guided knowledge transfer is introduced to supplement output learning with the teacher model’s intermediate features. The latency-constrained adaptive optimization module incorporates the target-latency requirement during training so that optimization considers both prediction error and actual inference efficiency. After training, the dynamic structured-sparsification module adjusts the structured sparsity level and briefly fine-tunes the selected student model according to runtime feedback from the target hardware to suppress groups of low-importance weights. Through these modules, LCDP integrates knowledge transfer, latency constraints, and post-training structured sparsification within a lightweight forecasting model for deployment.

### 3.2. Latency-Constrained Adaptive Optimization

Under conventional knowledge distillation, a student model improves its predictive capability mainly by fitting the teacher model’s outputs or intermediate features, but this process does not directly constrain actual inference latency on the target hardware platform. Consequently, even a student model with fewer parameters may fail to achieve the expected low latency in real-world deployment because of operator types, network structure, or memory-access patterns. We therefore design an LCAO mechanism that combines prediction error, distillation error, local feature-alignment error, and a latency-penalty term within a unified optimization objective. Rather than merely reducing parameter count, LCAO introduces a target-latency constraint during training so that student-model updates are jointly influenced by forecasting accuracy and inference efficiency, providing a more suitable basis for subsequent dynamic structured sparsification.

In LCAO, actual inference time on the target hardware platform contributes to model training through the latency-constraint term. Specifically, the student model’s inference latency is measured periodically during training and compared with a preset target. When the actual latency is below or close to the target, optimization is dominated by the prediction, distillation, and feature-alignment losses. When the actual latency exceeds the target range, the latency-penalty term exerts a greater influence on the total loss and guides parameter updates toward lower-latency inference. Unlike methods that reduce only parameter count or computational cost, LCAO focuses on actual runtime performance on the target hardware, allowing training to account for both prediction error and deployment efficiency. The student model can therefore retain its basic forecasting capability while gradually converging toward a parameter space that is better suited to deployment.

To further describe the optimization process of LCAO, let the student-model prediction be ${\hat{Y}}_{s}$, the teacher-model prediction be ${\hat{Y}}_{t}$, the ground-truth label be Y, and the intermediate features of the student and teacher models be $F_{s}$ and $F_{t}$. In this framework, the student-model training objective comprises the output-distillation loss, local feature-alignment loss, and latency-constraint penalty. The total loss is defined as follows:

(1) $$L_{total}=L_{kd}+\beta L_{feat}+\lambda_{lat}(e)L_{lat}$$

where $L_{kd}$ denotes the output-distillation loss, $L_{feat}$ denotes the local feature-guided feature-alignment loss, and $L_{lat}$ denotes the latency-constraint penalty. β is the weight of the feature-alignment loss, and $L_{lat}(e)$ is the latency-constraint weight that varies with the training epoch.

The output-distillation loss comprises a teacher-guided output-matching term and a hard-label prediction term, as follows:

(2) $$L_{kd}=\alpha_{s}{\left\|{\hat{Y}}_{s}-{\hat{Y}}_{t}\right\|}_{2}^{2}+\alpha_{h}{\left\|{\hat{Y}}_{s}-Y\right\|}_{2}^{2}$$

where $\alpha_{s}$ and $\alpha_{h}$ are the weights of the teacher-guided output-matching term and the hard-label prediction term, respectively. The former encourages the student model to approximate the teacher model’s predictions and transfer its predictive knowledge, whereas the latter preserves the student model’s ability to fit the ground-truth forecasting target.

The local feature-guided knowledge-transfer loss is defined as follows:

(3) $$L_{feat}=\frac{1}{N}\sum_{i=1}^{N}{\left\|F_{s}^{i}-\phi (F_{t}^{i})\right\|}_{2}^{2}$$

where ϕ(·) is a feature-mapping function that maps the teacher model’s intermediate features to the same dimensionality as the student features. This loss allows the student to use local temporal representations from the teacher while learning the final forecasts.

The target latency is defined as follows:

(4) $$T_{target}=\rho T_{t}$$

where $T_{t}$ is the teacher model’s measured inference latency on the target hardware platform, and ρ is the target-latency ratio.

The latency-constraint penalty is defined as follows:

(5) $$L_{lat}=\left\{\begin{array}{c} 0,T_{s}\leq (1+\delta )T_{target}\\ \eta (T_{s}-T_{target}),T_{s}>(1+\delta )T_{target} \end{array}\right.$$

where $T_{s}$ is the measured inference latency of the student model, δ is the latency-tolerance coefficient, and η is the latency-penalty coefficient. The discontinuity at $T_{s}=(1+\delta )T_{target}$ is intentional. The interval below this boundary is treated as a tolerance region to prevent minor fluctuations in hardware timing from activating the latency penalty. Once the measured latency exceeds the upper tolerance boundary, the penalty is calculated relative to $T_{target}$, rather than only to $(1+\delta )T_{target}$. This design immediately applies the full target-latency violation after the tolerance is exceeded, thereby imposing a stricter constraint on models that clearly fail to meet the deployment target.

To prevent the latency constraint from affecting the learning of predictive capability too early in training, we use a dynamic weighting strategy to adjust the strength of the latency-penalty term:

(6) $$\lambda_{lat}(e)=\left\{\begin{array}{c} 0,e<0.4E\\ \lambda_{0}\frac{e-0.4E}{0.4E},0.4E\leq e<0.8E\\ \lambda_{0},e\geq 0.8E \end{array}\right.$$

where e is the current training epoch, E is the total number of epochs, and $\lambda_{0}$ is the base weight of the latency constraint. This strategy allows the model to focus primarily on the forecasting target and teacher knowledge during early training and to progressively strengthen the influence of the latency constraint during the middle and later stages.

These equations show that LCAO jointly incorporates output distillation, local feature alignment, and the latency constraint into student-model training. The output-distillation loss maintains the student’s ability to fit the forecasting target, the local feature-alignment loss supplements the lightweight student’s temporal representations, and the latency-constraint term supplies deployment feedback based on inference time measured on the target hardware. By dynamically adjusting the weight of the latency constraint, LCAO focuses on learning predictive capability during early training and progressively strengthens the constraint on inference efficiency during the middle and later stages. This process allows the student model to reduce its dependence on high-latency computational paths while retaining its basic forecasting performance and provides a stable optimization basis for subsequent latency-guided structured sparsification.

### 3.3. Deployment-Oriented Dynamic Structural Pruning

After latency-constrained adaptive optimization, groups of low-importance weights may remain in the student model. To adapt the pruning level to the target platform, we design DOSP as a latency-guided structured-sparsification procedure. Unlike methods that use a single fixed pruning ratio, DOSP evaluates candidate ratios according to measured latency. For each candidate, group-wise L2-norm pruning is applied to the output groups of the student model’s fully connected layers, setting selected weight groups to zero while retaining the original dense layer dimensions.

DOSP searches over candidate pruning ratios rather than pruning only once at a fixed ratio. After LCAO training, the trained student weights are loaded for each candidate ratio, structured pruning is applied, and inference latency is measured on the target hardware. The ratio is progressively increased until the target latency is met or the predefined range is exhausted. If no candidate satisfies the target, the candidate with the lowest measured latency is retained.

The deployment constraint is expressed as follows:

(7) $$T_{s}(M^{\left(k\right)})\leq T_{target}$$

where $M^{\left(k\right)}$ denotes the student model obtained using the k-th candidate pruning ratio, and $T_{s}(M^{\left(k\right)})$ is its measured inference latency on the target hardware platform. When this condition is not satisfied, the next candidate pruning ratio is evaluated.

The different latency thresholds used in LCAO and DOSP reflect their distinct roles. During LCAO training, $(1+\delta )T_{target}$ provides a tolerance region that prevents minor fluctuations in hardware timing from repeatedly activating the latency penalty. By contrast, DOSP is a post-training, deployment-oriented structured-sparsification procedure, for which $T_{target}$ serves as the final acceptance criterion. Candidate pruning ratios are evaluated against the strict deployment target $T_{s}(M^{\left(k\right)})\leq T_{target}$; if none satisfy this condition, the candidate with the lowest measured latency is retained.

The pruning update is expressed as follows:

(8) $$M^{\left(k+1\right)}=P(M^{\left(k\right)},r_{k})$$

where P(·) denotes the structured pruning operation, $r_{k}$ is the pruning ratio used in the kth iteration. We progressively increase the pruning ratio to search for a structured sparsity level that satisfies the latency constraint.

Structured pruning sets groups of low-importance weights in the student model to zero and changes the original feature-propagation paths, which may affect the forecasts. DOSP therefore performs a short fine-tuning stage after each pruning iteration to update the sparsified student’s parameters and mitigate pruning-induced performance fluctuations. During fine-tuning, the teacher outputs and ground-truth labels remain training constraints, allowing the sparsified model to maintain relatively stable forecasting capability under the resulting sparsity pattern. LCAO and DOSP thus play complementary roles. LCAO introduces latency constraints and knowledge transfer during training to establish a suitable optimization basis for the student, whereas DOSP further adjusts the structured sparsity pattern according to actual inference feedback and improves the deployment adaptability of the sparsified model. Their combination allows LCDP to coordinate forecasting performance with practical inference efficiency.

In the present implementation, DOSP performs latency-guided structured sparsification by setting groups of low-importance weights associated with selected neurons to zero while retaining the original dense layer dimensions. Consequently, all saved ablation checkpoints contain 9416 registered parameters, of which 7336 form the active inference backbone, and 2080 belong to the training-only feature-alignment projection described in Section 3.1. Because this projection is not invoked during inference, all variants require 50,848 dense inference MACs, although the DOSP-enabled variants have different structured sparsity patterns. Therefore, the effect of DOSP is evaluated using forecasting accuracy and measured CPU latency rather than reductions in the registered parameter count or dense inference MACs. Physical graph compaction of the sparsified units is left to deployment-specific implementation.

### 3.4. Training and Deployment Pipeline

**Figure 2:**
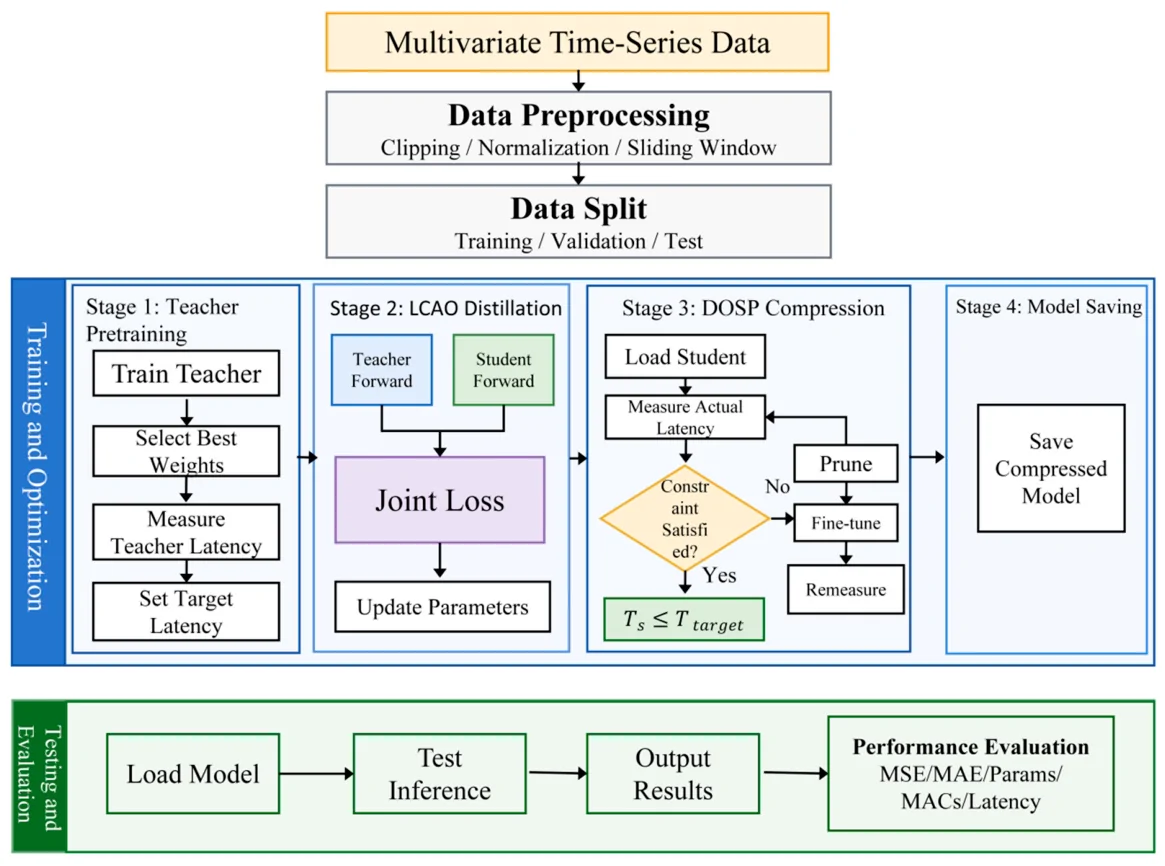
Training, structured sparsification, and deployment pipeline of the proposed LCDP framework.

To further describe the training, compression, and deployment of LCDP, we divide the overall pipeline into a training-and-optimization stage and a testing-and-deployment stage, as shown in Figure 2. The training-and-optimization stage comprises teacher-model pretraining, LCAO distillation training, DOSP dynamic structured sparsification, and saving the sparsified model. The testing-and-deployment stage comprises model loading, inference on the test set, performance evaluation, and deployment on the target platform. This pipeline presents the complete process from raw multivariate time-series input to deployment of the sparsified student model.

As shown in Figure 2, the LCDP pipeline comprises data preprocessing, training and optimization, and testing and deployment. First, the raw multivariate time series is processed through outlier clipping, normalization, and sliding-window construction and then divided chronologically into training, validation, and test sets. In the training-and-optimization stage, the teacher model is first pretrained, the best-performing weights are selected according to validation results, and teacher-model latency is measured to set the target latency. The student model is then trained via distillation using LCAO, with its parameters jointly updated by the output-distillation loss, feature-alignment loss, and latency-constraint term.

After distillation training, DOSP dynamically adjusts the structured sparsity level of the student model according to inference latency measured on the target hardware. If the model does not satisfy the latency requirement, structured pruning and short-term fine-tuning are performed, followed by another latency measurement. Once the model meets the latency requirement, the sparsified student is saved. During testing and deployment, the sparsified model is loaded and evaluated on the test set using MSE, MAE, parameter count, MACs, and actual inference latency, after which it can be used for low-latency forecasting on the evaluated CPU platforms.

## 4. Experiments and Results

### 4.1. Dataset Description and Experimental Setup

#### 4.1.1. Datasets

Experiments were conducted on the ETTh1, ETTm1, and ETTm2 subsets of the Electricity Transformer Temperature (ETT) dataset. The dataset records the operating data of electricity transformers at two sites from July 2016 to July 2018. The suffixes 1 and 2 denote different data-collection sites, whereas h and m indicate sampling intervals of 1 h and 15 min, respectively. ETTh1 contains 17,420 hourly time points, and ETTm1 and ETTm2 each contain 69,680 time points sampled at 15 min intervals, giving 156,780 time points across the three datasets. Each time point contains a date field and seven numerical variables. HUFL, HULL, MUFL, MULL, LUFL, and LULL represent six load variables of different levels and types, and OT denotes transformer oil temperature. We used a multivariate-input, univariate-output setting: historical observations of all seven numerical variables were used as model inputs, and OT was the forecasting target.

#### 4.1.2. Comparative Experiment Design

To comprehensively evaluate LCDP in terms of forecasting accuracy, model complexity, and inference efficiency, we selected representative multivariate time-series forecasting models as baselines. These models cover recurrent neural networks, attention mechanisms, lightweight forecasting, and sequence compression, including GRU, Transformer, Informer, BESN, DLinear, KAN, and Token Merging.

GRU, Transformer, and Informer represent deep temporal forecasting models of different complexity and were used to examine whether LCDP can maintain predictive capability comparable to high-performing forecasting models while reducing model overhead. BESN, DLinear, and KAN represent structurally simplified or low-complexity modeling methods and were used to compare the trade-offs between forecasting accuracy and computational efficiency under different lightweight strategies. Token Merging represents efficient computation based on sequence-representation compression and was used to compare input-level compression with the proposed dynamic structural optimization in terms of efficiency and accuracy. Together, these baselines enable evaluation of LCDP along three dimensions: forecasting performance, model size, and inference latency.

To evaluate model deployment efficiency across different CPU hardware platforms, CPU inference latency was measured on both a local laptop and a cloud server. The local platform (CPU 1, Honor Device Co., Ltd., Shenzhen, Guangdong, China), which was used for the original experiments, was equipped with an Intel Core Ultra 5 225H processor and provided 14 logical processors. The server platform (CPU 2, Shanghai Gengcong Information Technology Co., Ltd., Shanghai, China), which was used for the additional hardware evaluation, was equipped with an Intel Xeon Gold 6133 @ 2.50 GHz and was allocated eight logical processors, corresponding to four cores with two threads per core. All latency measurements were performed in CPU-only mode without GPU acceleration. The model architectures, input configurations, and evaluation procedures were kept identical across the two platforms. The latency results obtained on the two platforms are reported separately to evaluate inference efficiency under different computing environments. Results are reported in Section 4.2 from separate training and evaluation runs conducted on CPU 1 and CPU 2, respectively. Accordingly, only the inference latency values are platform-specific, while the small differences in MSE and MAE arise from run-to-run variation between the independently trained models. The ablation results are presented in Section 4.3.

To reduce timing fluctuations, CPU inference latency on both hardware platforms was measured after 500 warm-up iterations. The timing procedure was repeated 20 times, with 500 forward passes performed in each repetition. The per-pass latency was first averaged within each repetition, and the latency valuesare reported as the mean ± standard deviation of the 20 repetition-level averages, corresponding to 10,000 timed forward passes for each model-dataset combination. MSE and MAE were computed on the complete test set using the corresponding trained checkpoint and identical evaluation settings. Because each model was trained once on each platform, the accuracy metrics reported for each platform are point estimates rather than averages with standard deviations across repeated training runs.

All models used a multivariate-input, univariate-output setting, with both the historical input length and forecasting horizon set to 96. The inputs comprised seven numerical variables, and OT was the forecasting target. The data were divided chronologically into training, validation, and test sets. The learning rate, number of epochs, and structural parameters of each model were selected according to convergence on the validation set, as listed in Table 1.

For LCDP, the reported 9416 parameters include all registered trainable parameters in the saved student checkpoint: 7336 parameters belong to the active inference backbone, whereas 2080 parameters belong to the 64 → 32 feature-alignment projection used only during training. The projection remains stored in the checkpoint but is not executed during inference.

To improve the reproducibility of the proposed method, the LCDP-specific hyperparameters and teacher-model statistics are summarized in Table 2.

The teacher-model latencies were measured on CPU 1 (Intel Core Ultra 5 225H) and were used to determine the dataset-specific target latencies according to $T_{target}=\rho T_{t}$. Because the teacher model is used only during training, it is not included in the model-complexity comparisons for final inference.

#### 4.1.3. Ablation Experiment Design

To verify the effectiveness of the constituent modules in LCDP and analyze how different optimization mechanisms affect forecasting performance and deployment efficiency, we conducted ablation experiments on ETTh1, ETTm1, and ETTm2. LCDP contains three core modules: LCAO, Local Feature Distillation (LFD), and DOSP. The ablation experiments addressed the following three questions:

(1) Can latency-constrained adaptive optimization improve the balance between forecasting accuracy and inference efficiency?

Conventional lightweight forecasting models generally minimize prediction error as their primary objective and ignore inference efficiency in the target runtime environment. LCAO introduces a latency constraint during training so that optimization accounts for both forecasting performance and deployment efficiency. To evaluate LCAO, we compared the Base model with Base + LCAO and examined the changes in prediction error and inference latency after introducing the latency constraint.

(2) Can local feature distillation improve the temporal representational capacity of a lightweight student model?

Because model compression reduces network capacity, lightweight models may be less capable of modeling complex nonlinear variations and local fluctuation patterns. LFD guides the student model to learn local temporal representations and intermediate features from the teacher, strengthening the representation of important change patterns. We compared Base with Base + LFD and also examined combination models containing LFD to analyze the effect of local feature transfer on forecasting performance.

(3) Can dynamic structured sparsification further reduce deployment overhead?

Conventional pruning methods generally determine sparsity using a fixed ratio or preset threshold and therefore have limited adaptability to different deployment environments. DOSP instead selects a structured sparsity pattern according to measured latency feedback from the target hardware while retaining the original dense layer dimensions. We compared the actual inference latency and forecasting performance of Base and Base + DOSP to evaluate the contribution of latency-guided structured sparsification to deployment efficiency.

We further constructed the complete LCDP model to evaluate the coordinated optimization achieved by LCAO, LFD, and DOSP. All ablation experiments used the same data preprocessing, data-splitting strategy, and training hyperparameters. The variants were executed sequentially after a single global random seed had been set; consequently, the random state continued to advance, and the variants did not reuse identical initial weights or minibatch orders. For the A0/A1 verification discussed in Section 4.3.1, we additionally performed paired runs using identical initialization and minibatch order to isolate the effect of LCAO. The evaluation metrics included MSE and MAE for prediction error and parameter count (Params), multiply-accumulate operations (MACs), and CPU inference latency (Latency) for deployment efficiency.

The ablation configurations are listed in Table 3. Base denotes a lightweight student model trained using output-layer knowledge distillation only and serves as the reference for evaluating the LCDP optimization modules. LCAO, LFD, and DOSP were added individually to analyze their independent contributions, and the Full LCDP model was used to evaluate the overall performance obtained by jointly applying all three modules.

### 4.2. Comparative Experimental Results

To compare model performance in multivariate time-series forecasting, we conducted experiments on ETTh1, ETTm1, and ETTm2 and analyzed the results from three perspectives: forecasts, step-wise absolute errors, and aggregate evaluation metrics. Forecast curves were used to examine how well each model tracked the overall variation and local fluctuations of the ground-truth series. Absolute-error plots show the distribution of errors across time steps. The aggregate metrics compare forecasting accuracy, structural scale, and inference efficiency using MSE, MAE, parameter count, MACs, and inference latency. The results are discussed below in the order ETTh1, ETTm1, and ETTm2.

**Figure 3:**
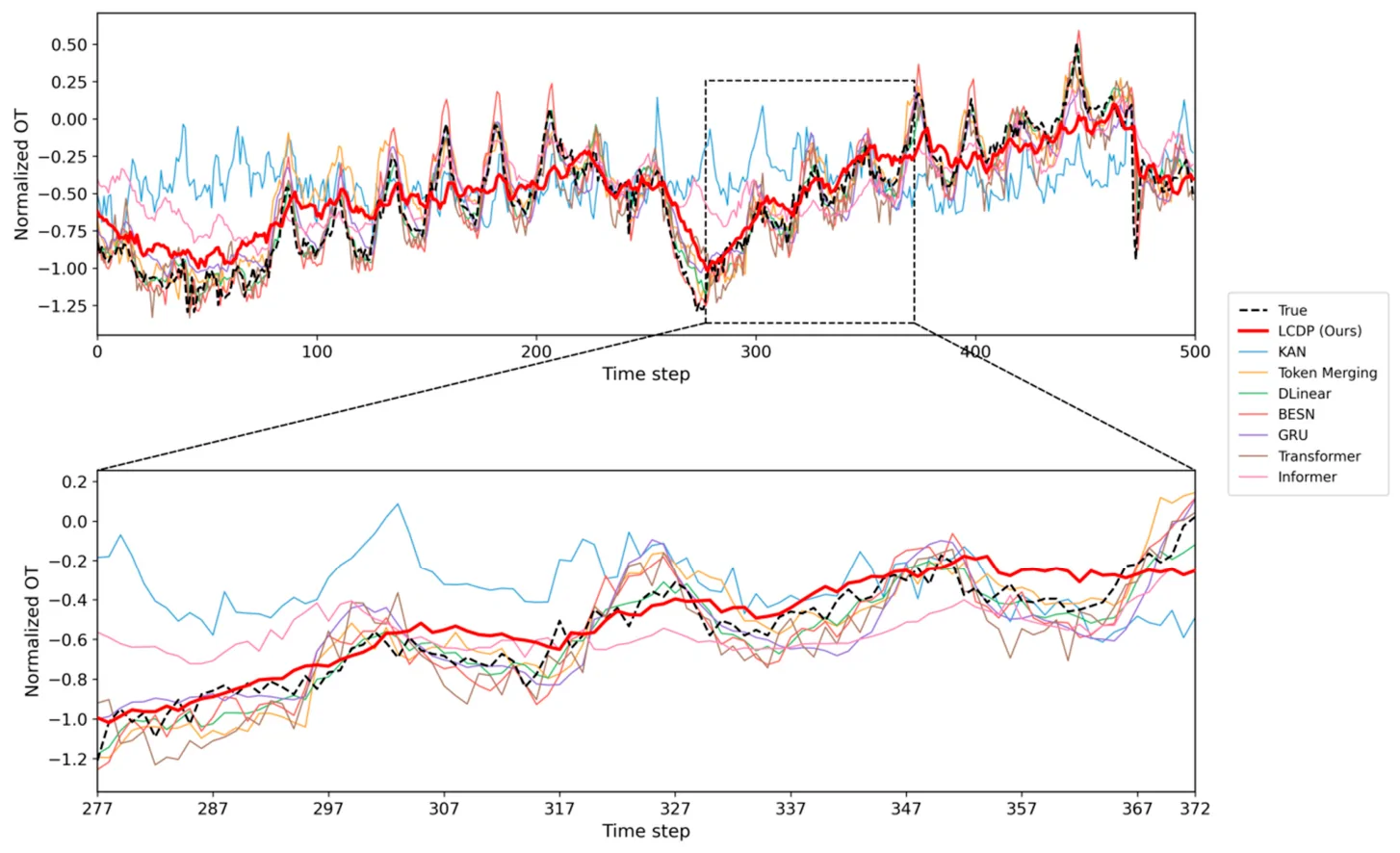
Forecasting results of different models on the ETTh1 test set.

**Figure 4:**
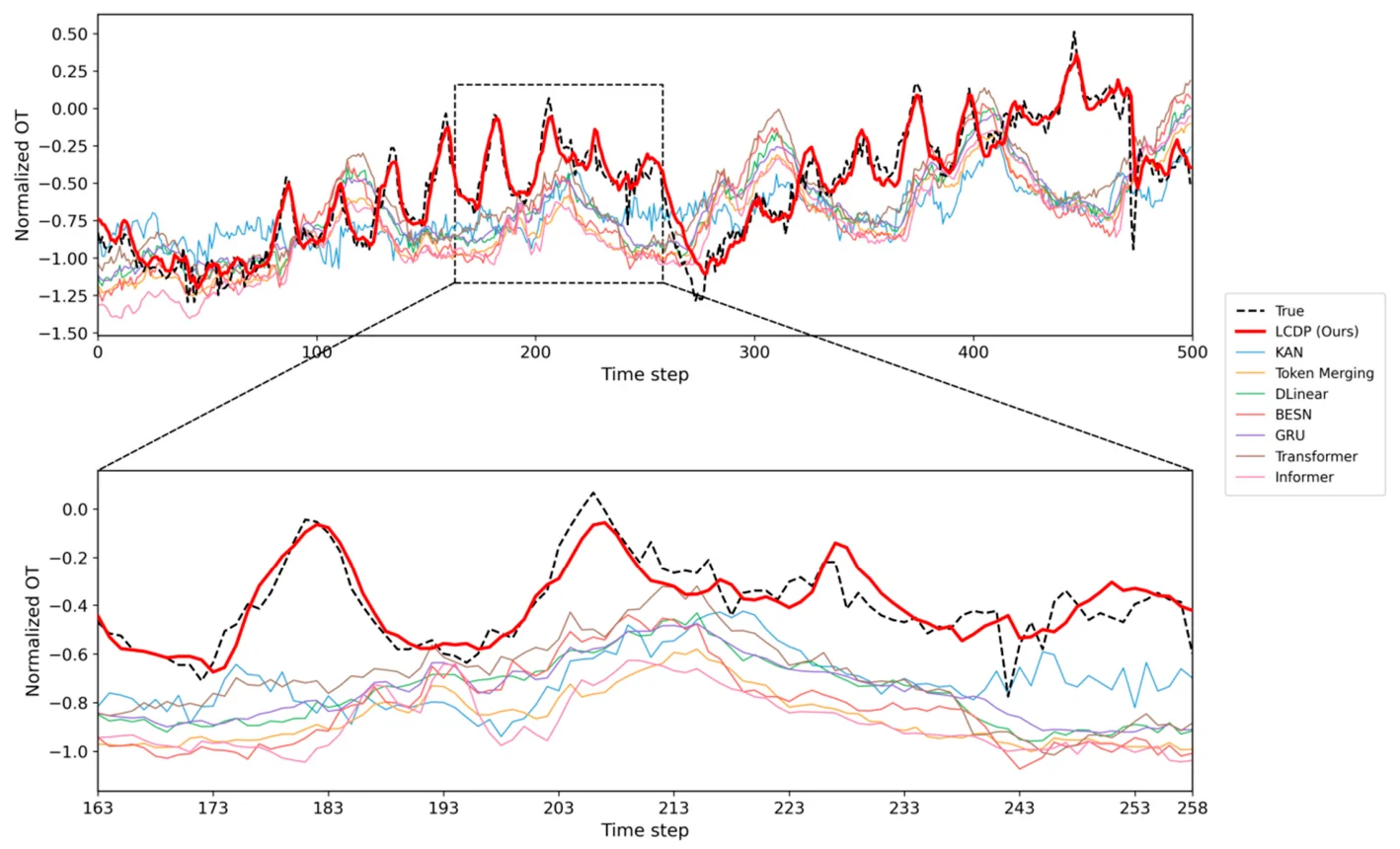
Forecasting results of different models on the ETTm1 test set.

**Figure 5:**
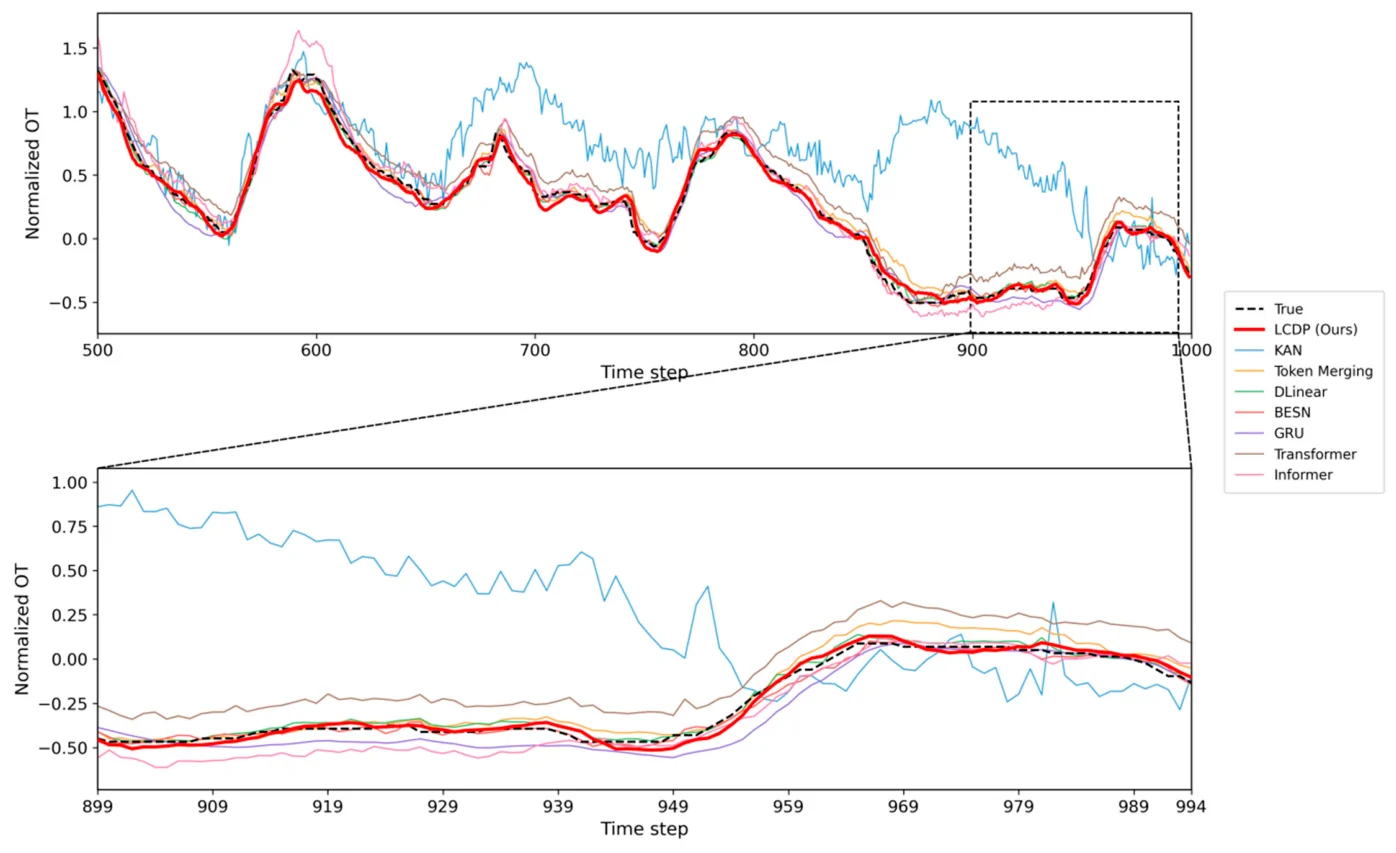
Forecasting results of different models on the ETTm2 test set.

Figure 3 shows the forecasts over the first 500 time steps of the ETTh1 test set. Figure 4 shows the corresponding forecasts for the ETTm1 test set. Figure 5 shows the forecasts over time steps 500–999 of the ETTm2 test set. Corresponding local intervals are enlarged for closer inspection.

Figure 3, Figure 4 and Figure 5 show that all models generally tracked the overall trends of the three datasets, although their performance differed around local peaks, troughs, and turning points. The LCDP forecasts remained close to the ground-truth series overall and consistently captured variations in the series within the enlarged local intervals. Compared with several baselines, LCDP showed more stable forecasting behavior across the datasets, indicating reliable trend tracking and local forecasting capability.

**Figure 6:**
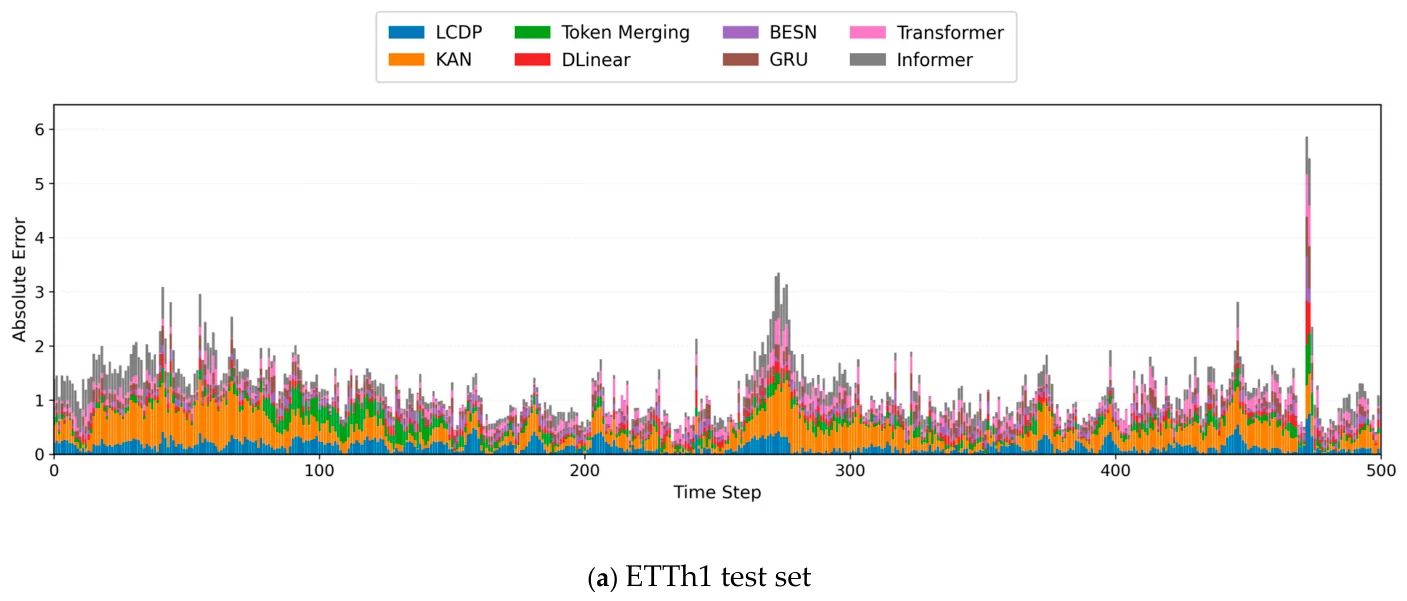

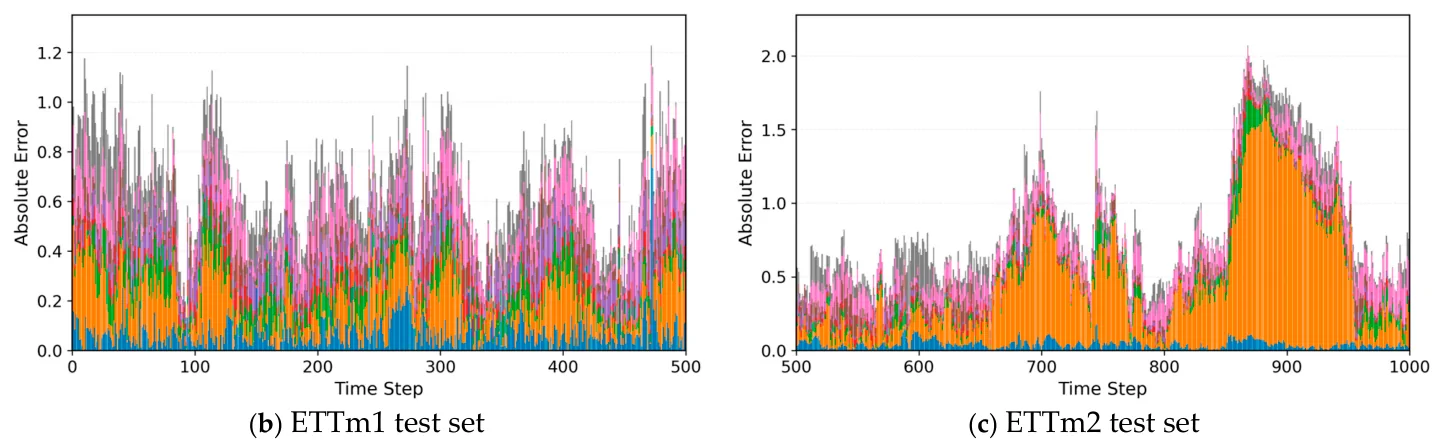
Step-wise absolute forecasting errors of different models on the ETTh1, ETTm1, and ETTm2 test sets.

To further compare step-wise prediction errors during testing, Figure 6 combines the absolute errors on ETTh1, ETTm1, and ETTm2. The upper subplot shows the absolute error over time steps 0–499 of the ETTh1 test set, the lower-left subplot shows time steps 0–499 of the ETTm1 test set, and the lower-right subplot shows time steps 500–999 of the ETTm2 test set. The horizontal axis denotes the test time step, the vertical axis denotes the absolute prediction error, and different colors represent different models.

As shown in Figure 6, the absolute errors of all models fluctuated over time and generally increased around pronounced changes in the series. Compared with several baselines, LCDP maintained relatively low and stable errors within the local intervals of all three datasets and did not exhibit sustained large deviations. The error distributions were consistent with the preceding forecast curves, indicating stable local forecasting performance across the datasets.

For a quantitative comparison of forecasting accuracy and inference efficiency, Table 4 and Table 5 summarize the parameter count and MACs of each model, together with its MSE, MAE, and CPU inference latency on ETTh1, ETTm1, and ETTm2. MSE and MAE quantify prediction error, parameter count and MACs reflect model size and computational cost, and CPU inference latency measures inference efficiency in the actual runtime environment.

For LCDP, the Params values reported in Table 4 and Table 5 denote all registered parameters in the saved student checkpoint. The active inference path contains 7336 parameters, whereas the remaining 2080 parameters belong to the training-only feature-alignment projection and are not executed during inference.

Table 4 shows that LCDP maintained low prediction errors on all three datasets while requiring relatively few MACs and achieving low actual inference latency. Although some baselines performed better on a single metric, such as KAN in parameter count and Dlinear in latency on ETTm1, LCDP provided a more stable overall balance among forecasting accuracy, computational cost, and inference efficiency.

Table 5 shows that LCDP achieved the lowest inference latency on CPU 2 across all three datasets while maintaining competitive forecasting accuracy and requiring relatively few MACs. Although Token Merging achieved slightly lower prediction errors on ETTh1 and Dlinear obtained a marginally lower MAE on ETTm2, LCDP consistently provided a favorable balance among forecasting accuracy, computational cost, and inference efficiency. These results further demonstrate that the efficiency advantage of LCDP remains stable across different CPU hardware platforms.

**Figure 7:**
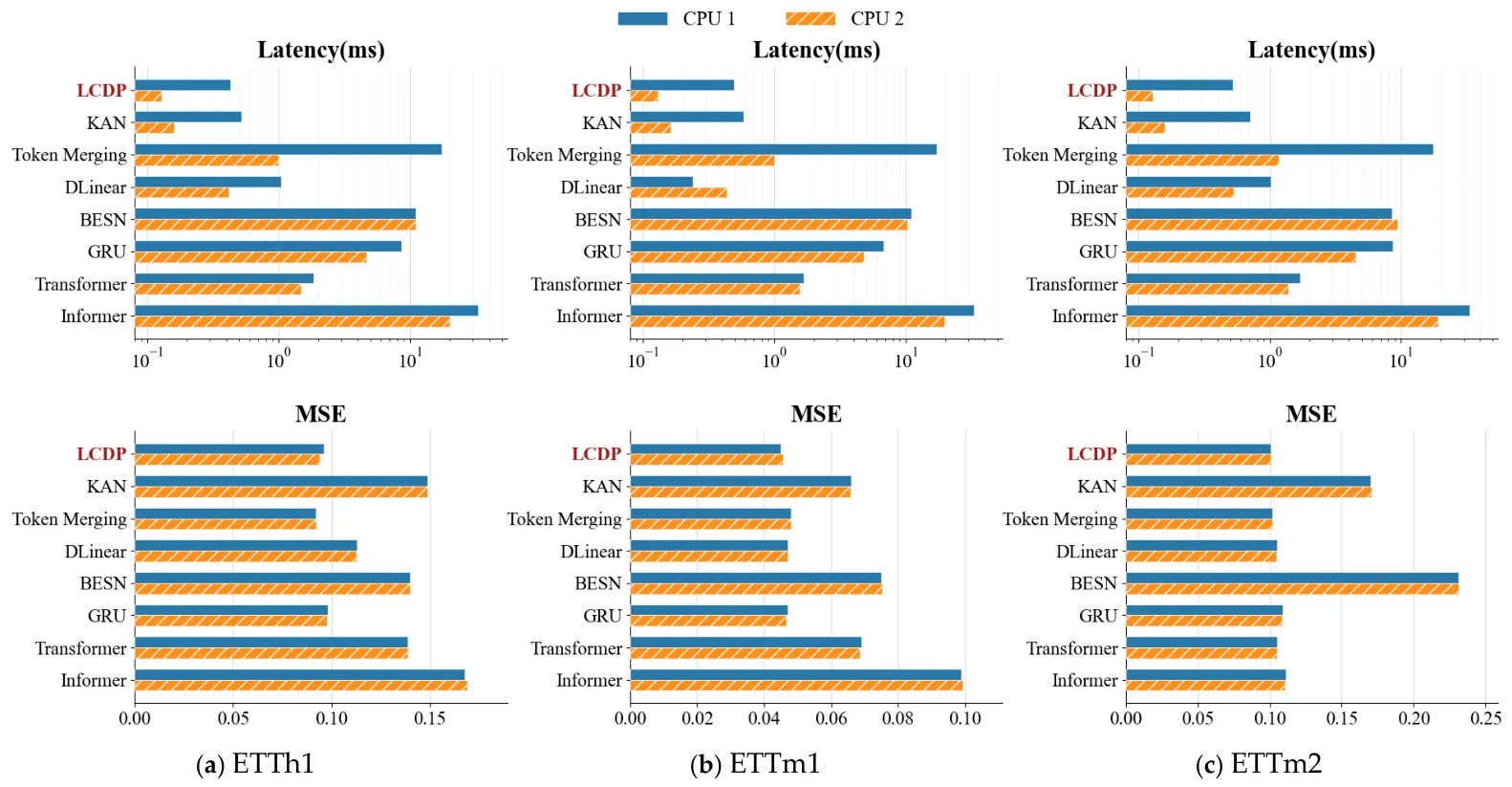
Cross-platform comparison of CPU inference latency and forecasting error (MSE) on the ETTh1, ETTm1, and ETTm2 test sets (from left to right). Latency is presented on a logarithmic scale.

Figure 7 shows that MSE remained nearly unchanged across the two CPUs, whereas inference latency varied substantially. On CPU 2, LCDP achieved the lowest latency on all three datasets while maintaining competitive accuracy, demonstrating stable cross-platform efficiency.

**Figure 8:**
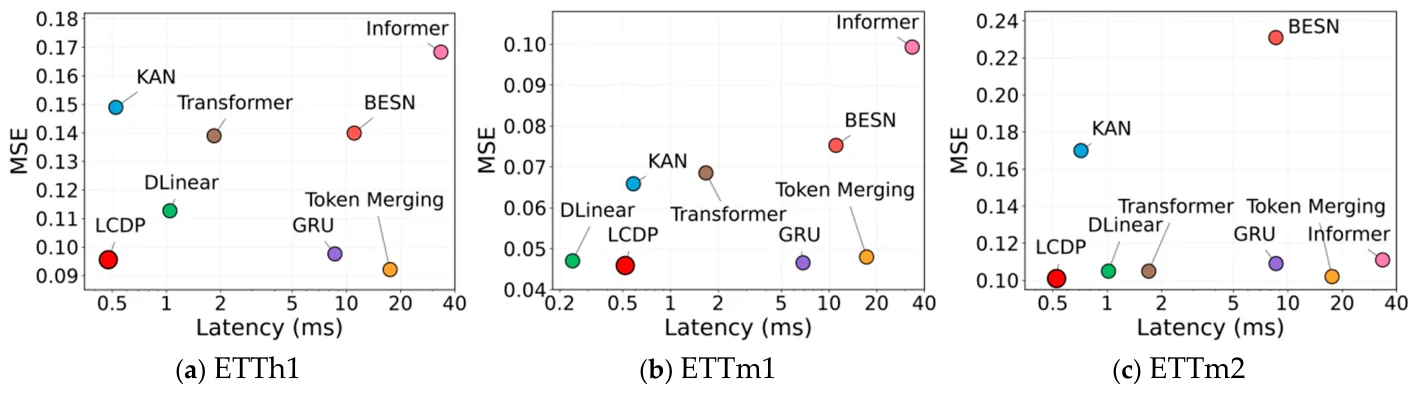
MSE–latency trade-offs of different models on the ETTh1, ETTm1, and ETTm2 test sets.

To visualize the trade-off between forecasting accuracy and practical inference efficiency, Figure 8 shows the two-dimensional MSE-latency distributions on ETTh1, ETTm1, and ETTm2.

Figure 8 shows a clear trade-off between prediction error and inference latency across the models; positions closer to the lower-left region generally indicate better overall performance. LCDP was located relatively close to the lower-left region on all three datasets, combining low prediction error with short actual inference latency. Although some models performed better on an individual metric, LCDP maintained a stable overall accuracy-efficiency balance.

### 4.3. Ablation Experiment Results

#### 4.3.1. Effect of Individual Modules

To analyze the effects of LCAO, LFD, and DOSP on model performance, we used the Base model (A0), which employs output-layer knowledge distillation only, as the ablation reference. The three modules were then introduced separately to form A1, A2, and A3, and their independent contributions were evaluated through changes in prediction error and inference latency. Table 6 and Table 7 also include the complete model A7 to compare the single-module variants with the full configuration and assess their coordinated gains.

All saved ablation checkpoints contain 9416 registered parameters, of which 7336 form the active inference path and 2080 belong to the training-only feature-alignment projection. Because the projection is not executed during inference, all variants require 50,848 dense inference MACs. The DOSP-enabled variants differ in their structured sparsity patterns rather than in the dense tensor dimensions.

To make the small differences among the ablation variants visible, the MSE and MAE values in Table 6 and Table 7 are reported to five decimal places. Even at this precision, the differences on ETTm1 and ETTm2 remain small. Accordingly, these results are interpreted as maintaining comparable forecasting accuracy rather than demonstrating substantial accuracy improvements.

Although A0, A1, and A2 share the same inference architecture and theoretical computational complexity, their measured latency differences were not uniformly within the repetition-level variability. On ETTh1, the A1-A0 and A2-A0 differences were 0.034 and 0.093 ms, exceeding the corresponding combined standard deviations of 0.030 and 0.048 ms. On ETTm1 and ETTm2, their absolute differences were only 0.004–0.011 ms and 0.001–0.008 ms, respectively, and remained within the combined measurement variability. Compared with A0, A7 reduced the mean latency by 0.022, 0.066, and 0.024 ms on ETTh1, ETTm1, and ETTm2, respectively. The reductions on ETTh1 and ETTm2 were smaller than the corresponding combined standard deviations of 0.032 and 0.027 ms, whereas the reduction on ETTm1 slightly exceeded the combined standard deviation of 0.055 ms. These results should therefore be interpreted as measured runtime tendencies rather than uniform evidence of structural acceleration.

Table 6 shows that the accuracy changes were not monotonic when the three modules were introduced individually, because each module serves a different optimization purpose and its effect depends on the temporal characteristics of the dataset. Compared with A0, the original single-run A1 result on ETTh1 showed higher MSE and MAE, while its measured latency also increased. To determine whether this difference was caused by the latency constraint, we repeated the A0/A1 comparison using identical initialization and minibatch order and recorded the student latency at every training epoch. The measured latency remained below the upper tolerance boundary throughout training; therefore, the latency-penalty term remained zero. Under this paired setting, A0 and A1 converged to identical checkpoints and produced identical forecasting metrics. The ETTh1 difference in the original single-run results is therefore consistent with run-to-run stochastic variation rather than an effect of an active LCAO penalty. The identical A0 and A1 forecasting metrics originally reported on ETTm1 and ETTm2 are also consistent with an inactive latency-penalty term. The corresponding small measured latency differences should be interpreted in light of runtime measurement variability. A2 produced small and inconsistent accuracy changes: both MSE and MAE increased on ETTh1, whereas the changes on ETTm1 and ETTm2 remained within a narrow range. A3 applied DOSP to induce a structured sparsity pattern, which may suppress either redundant or informative connections depending on the dataset, resulting in dataset-dependent accuracy changes. These results indicate that adding an individual module does not necessarily produce a uniform reduction in prediction error, since the three modules optimize different aspects of the model.

#### 4.3.2. Effect of Module Combination

Building on the single-module ablations, we evaluated three two-module combinations: A4 (LCAO + LFD), A5 (LCAO + DOSP), and A6 (LFD + DOSP). The complete model A7, which integrates all three modules, was included as an overall reference. Prediction error and inference latency were compared with the corresponding single-module variants and A7 to analyze complementarity and coordination among the modules and to assess the differences between the two-module combinations and the complete framework. The results are presented in Table 7.

The two-module results further explain why the accuracy changes were not monotonic. In A4, the combined configuration produced favorable accuracy-latency results on ETTh1. On ETTm1, A4 maintained comparable forecasting accuracy and showed lower measured latency than A1 and A2, although this latency difference should be interpreted in light of the runtime variability discussed above. By contrast, A5 showed increased prediction error on ETTh1. Given that the LCAO penalty remained inactive in the paired ETTh1 verification and that the original variants were obtained from independent training runs, this difference cannot be attributed specifically to the joint action of LCAO and DOSP. Moreover, structured sparsification does not necessarily translate directly into lower measured latency because the current implementation retains the original dense layer dimensions, and runtime is also affected by operator execution and hardware compatibility, which may account for the absence of consistent latency gains on ETTm1 and ETTm2. A6 achieved a more stable result, suggesting that LFD may compensate for part of the representational loss caused by DOSP. Finally, A7 combines the three components: LCAO, LFD, and DOSP. In the paired ETTh1 A0/A1 verification, the LCAO penalty remained inactive under the evaluated threshold; therefore, the original ETTh1 differences cannot be interpreted as evidence of an isolated LCAO effect. The slightly higher ETTh1 MSE of A7 relative to A4 and A6 should be interpreted cautiously because these variants were obtained from independent training runs; this difference cannot be attributed directly to a latency-accuracy trade-off.

#### 4.3.3. Comprehensive Analysis of LCDP

**Figure 9:**
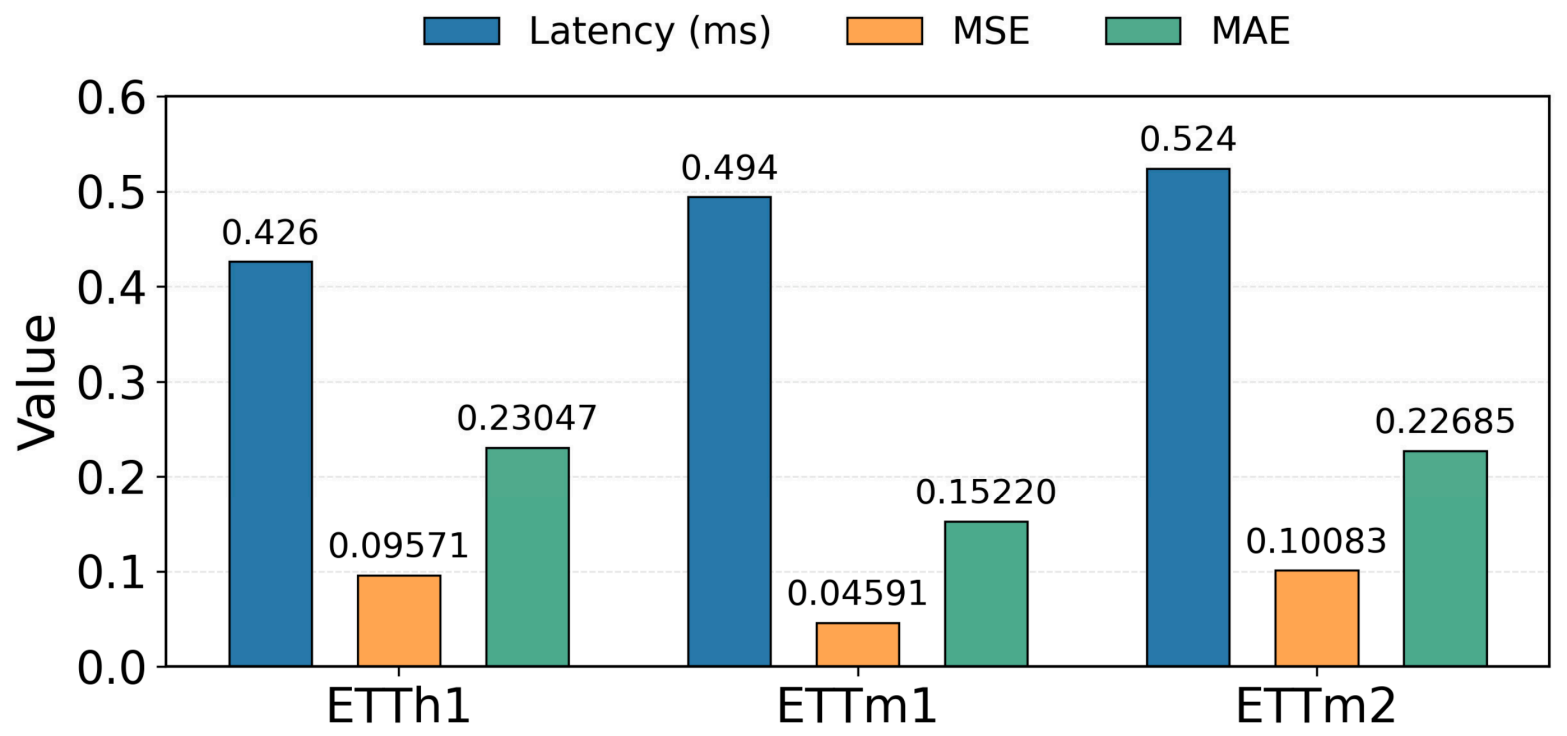
Forecasting accuracy and inference latency of the complete LCDP model on the ETTh1, ETTm1, and ETTm2 test sets.

Based on the preceding single-module and module-combination experiments, we further analyzed the overall performance of the complete LCDP model (A7). LCAO, LFD, and DOSP were jointly introduced to evaluate the model in terms of forecasting accuracy and inference efficiency. Figure 9 shows the latency, MSE, and MAE of A7 on ETTh1, ETTm1, and ETTm2.

Table 6 and Table 7, together with Figure 9, show that the complete LCDP model (A7) maintained low prediction error and low mean CPU inference latency across all three datasets. However, its latency reductions relative to A0 on ETTh1 and ETTm2 were within the combined measurement variability discussed in Section 4.3.1, and its measured latency on ETTm2 was comparable to that of A6. A7 retained the same registered checkpoint parameter count and dense inference MAC count as the other ablation variants because the current DOSP implementation applies structured group-wise sparsification without physically compacting the layer dimensions. The effect of DOSP is therefore evaluated using forecasting accuracy and measured latency rather than reductions in the registered checkpoint parameter count or dense inference MACs. Taken together, these results support a favorable overall accuracy-efficiency trade-off for A7 rather than a uniform latency advantage over every ablation variant.

**Figure 10:**
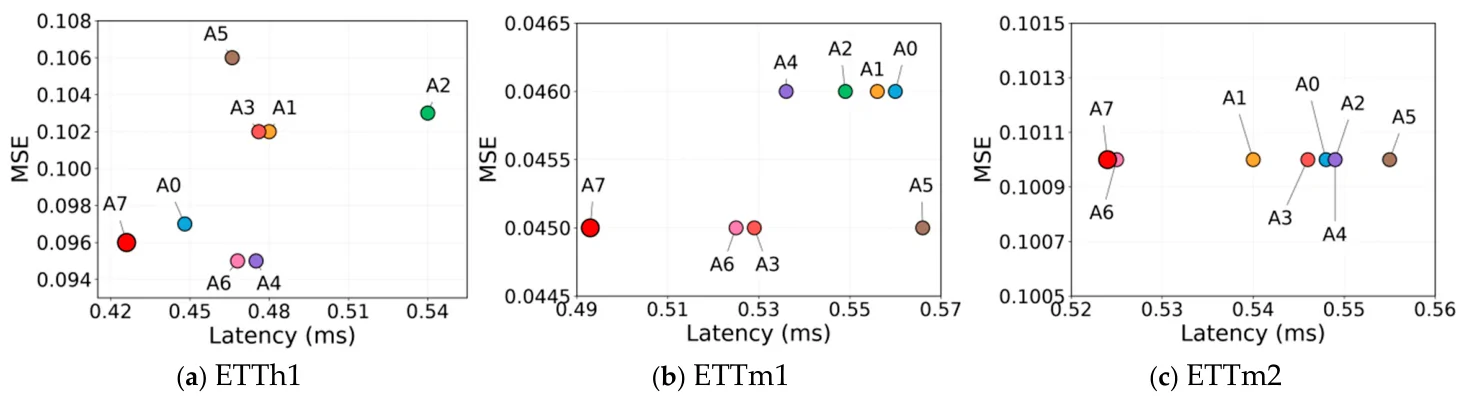
Accuracy-efficiency trade-offs of the ablation variants on the three ETT datasets.

To further visualize the relative relationships between forecasting accuracy and actual inference efficiency across the ablation variants, Figure 10 shows the two-dimensional MSE-latency distributions of A0–A7 on ETTh1, ETTm1, and ETTm2.

Figure 10 shows that the ablation variants exhibited different trade-offs between prediction error and inference latency. A7 maintained low MSE and low mean inference latency across the three datasets, providing a favorable overall accuracy-efficiency balance. Given the measurement variability discussed above, these distributions illustrate relative accuracy-latency trade-offs rather than demonstrating that A7 was uniformly faster than every other variant. Together with the preceding results, these observations indicate that the complete A7 configuration yields a favorable overall balance, although the paired ETTh1 verification did not show an isolated contribution from LCAO under the evaluated latency threshold.

## 5. Discussion

### 5.1. Effectiveness of the Proposed Framework

The overall experimental results show that LCDP maintained relatively stable forecasting performance on ETTh1, ETTm1, and ETTm2 across different forecasting intervals and reduced actual CPU inference latency while achieving MSE and MAE values comparable to or lower than those of existing models. The comparative and ablation experiments further indicate that latency-constrained adaptive optimization allows training to account for both prediction error and deployment efficiency, whereas DOSP determines a structured sparsity pattern after training by evaluating candidate pruning ratios using measured latency on the target hardware. Their combination improved practical inference efficiency while largely maintaining forecasting accuracy. LCDP therefore achieved a reasonable balance between forecasting accuracy and actual runtime latency, indicating coordination between latency-constrained optimization and post-training structured sparsification.

The ablation results show that the effects of the individual modules were dataset-dependent and non-monotonic rather than uniformly beneficial. For LCAO, the paired ETTh1 A0/A1 verification showed that the latency-penalty term remained inactive under the evaluated threshold. With identical initialization and minibatch order, A0 and A1 converged to identical checkpoints and produced identical forecasting metrics, indicating that the difference in the original ETTh1 single-run results was consistent with training stochasticity and was not attributable to an active LCAO penalty. The identical A0 and A1 metrics originally reported on ETTm1 and ETTm2 are also consistent with an inactive latency-penalty term. DOSP likewise produced small and dataset-dependent changes and did not consistently reduce measured latency because the current implementation retains the original dense layer dimensions. Nevertheless, the complete LCDP model maintained low mean CPU inference latency on all three datasets while largely preserving forecasting accuracy. Its latency reductions relative to A0 on ETTh1 and ETTm2 remained within the combined measurement variability, and its measured latency on ETTm2 was comparable to that of A6. These results indicate that the complete framework provides a favorable overall accuracy-efficiency balance under the evaluated configuration, although no isolated contribution from LCAO was observed in the paired ETTh1 verification.

### 5.2. Efficiency–Accuracy Trade-Off and Module Interpretation

Across forecasting accuracy, parameter count, MACs, and inference latency, LCDP does not merely minimize model size or theoretical computation. Instead, it balances prediction error, model complexity, and runtime efficiency for the actual hardware environment. The experiments show that fewer parameters or MACs do not necessarily correspond to lower practical inference latency; model structure, operator execution, and hardware compatibility also affect runtime efficiency. Parameter count and theoretical computational cost alone therefore cannot fully represent the practical deployability of a lightweight model. By including measured latency in the optimization objective and using DOSP after training to select a fixed structured sparsity pattern for deployment, LCDP achieved low measured CPU inference latency while maintaining competitive forecasting accuracy, thereby narrowing the gap between theoretical lightweight indicators and actual runtime efficiency.

### 5.3. Practical Implications, Limitations, and Future Work

The proposed method is intended for time-series forecasting scenarios with limited computing resources and response time requirements, such as edge-computing nodes, industrial equipment monitoring, and online sensor-data analysis. In these scenarios, a model must retain basic forecasting capability while satisfying constraints on device computing power, storage capacity, and real-time response. LCDP incorporates actual inference latency into the training constraints and uses DOSP after training to select a structured sparsity pattern for deployment, aligning model optimization more closely with the target hardware environment. This design indicates that directly incorporating deployment efficiency into the optimization objective and combining it with latency-guided post-training structured sparsification can provide a practical approach to efficient time-series forecasting in resource-constrained environments.

This study has several limitations. The current optimization process focuses primarily on prediction error and CPU inference latency and does not explicitly consider device energy consumption or peak memory usage during operation. Although the additional experiments evaluated inference latency on two CPU platforms, deployment performance across a broader range of heterogeneous hardware platforms, including edge processors and GPUs, requires further validation under more complex resource-constrained conditions. In addition, the current dynamic compression strategy uses a relatively fixed range of structural adjustments and has limited adaptability to different data characteristics and hardware conditions. Future work could incorporate energy consumption, memory usage, computational cost, and inference latency into a multi-objective resource-aware optimization framework and investigate more adaptive structural search and dynamic routing mechanisms, enabling the model to select an appropriate execution structure according to task characteristics and hardware conditions.

## 6. Conclusions

This study proposed a Latency-Constrained Distillation and Dynamic Pruning (LCDP) framework for efficient multivariate time-series forecasting under resource and latency constraints. LCDP integrates latency-constrained adaptive optimization, local feature-guided knowledge transfer, and deployment-oriented dynamic structural pruning into a unified training and deployment pipeline. By incorporating hardware-measured inference latency into model optimization and dynamically adjusting the structured sparsity level of the student model, the proposed framework jointly considers forecasting accuracy and practical runtime efficiency.

Experiments on the ETTh1, ETTm1, and ETTm2 datasets showed that LCDP achieved competitive forecasting accuracy with a compact model structure and low CPU inference latency. The results also demonstrated that theoretical complexity measures, such as parameter count and MACs, do not necessarily reflect practical inference efficiency. The ablation studies showed that the complete A7 configuration provided a favorable accuracy-latency trade-off while largely preserving forecasting performance. However, the paired ETTh1 A0/A1 verification did not show an isolated contribution from LCAO under the evaluated latency threshold, and the latency reductions relative to A0 on ETTh1 and ETTm2 remained within the combined measurement variability. The measured latency of A7 was also comparable to that of A6 on ETTm2.

Although the additional CPU experiments provide an initial assessment of cross-platform performance, the current study focuses primarily on CPU inference latency and does not explicitly consider energy consumption or memory usage. The present evaluation is also limited to three ETT benchmarks from the same application domain. Future work will extend LCDP to multi-objective resource-aware optimization and evaluate its robustness and generalizability across broader hardware platforms and diverse forecasting domains.

## Figures and Tables

**Table 1:** Model configurations, training settings, and parameter counts used in the comparative experiments.

Model	Selection Rationale	Core Structural Configuration	Batch/Epoch/LR	Trainable Parameters
GRU	Conventional temporal modeling	1 layer, hidden = 128	64/10/1 ${\times \;10}^{-3}$	64,992
Transformer	Complex dependency modeling	d=64, h=4, $L=2,d_{ff}=$ 256	64/10/5 ${\times \;10}^{-4}$	696,544
Informer	Long-sequence forecasting	d=512, h= 8, $L_{e/d}=2/1,d_{ff}=$ 2048	32/10/1 ${\times \;10}^{-4}$	11,325,953
BESN	Classical lightweight model	$N_{r}\;$= 500, ρ=0.9, s=0.2	64/10/5 ${\times \;10}^{-3}$	48,096
DLinear	Low-complexity forecasting	kernel = 25	64/10/5 ${\times \;10}^{-3}$	18,632
KAN	Nonlinear representation	2 layers, hidden = 64	64/25/5 ${\times \;10}^{-4}$	4609
Token Merging	Computational optimization	hidden = 64, $r_{drop}\;$= 16	64/25/5 ${\times \;10}^{-4}$	15,592
LCDP	Proposed method	teacher/student = 64/32; feature-alignment projection = 64 → 32	64/25 + 25 + 2/5 ${\times \;10}^{-4}$	9416

Note: Batch, Epoch, and LR denote the batch size, number of training epochs, and learning rate, respectively. For LCDP, the three epoch values correspond to teacher-model training, student-model training, and post-pruning fine-tuning. For BESN, Table 1 reports the number of trainable parameters, whereas Section 4.2 report the total parameter count, including the fixed reservoir.

**Table 2:** LCDP-specific hyperparameter settings and teacher-model statistics.

Category	Symbol or Dataset	Value	Description
Hyperparameters	ρ	1.05	Target-latency ratio
Hyperparameters	δ	0.10	Latency-tolerance coefficient
Hyperparameters	η	0.50	Latency-penalty coefficient
Hyperparameters	$\lambda_{0}$	0.005	Base latency-constraint weight
Hyperparameters	β	0.01	Feature-alignment loss weight
Hyperparameters	$\alpha_{s}$	0.85	Teacher-guided output-matching weight
Hyperparameters	$\alpha_{h}$	0.15	Hard-label prediction weight
Teacher model	Hidden dimension	64	Teacher representation dimension
Teacher model	Parameters	16,616	Number of trainable parameters
Teacher model	Network type	Feed-forward MLP	Channel-independent temporal encoder with a cross-channel output projection
Teacher model	Layer configuration	96 → 64 → 64 → 96; 7 → 1p	Three temporal fully connected layers and one cross-channel projection; ReLU is applied after the first two layers
Teacher model	Input/output setting	96/96	Seven input variables and one forecasting target (OT)
Teacher latency	ETTh1	0.535 ms	$T_{target}$ = 0.562 ms
Teacher latency	ETTm1	0.504 ms	$T_{target}$ = 0.529 ms
Teacher latency	ETTm2	0.507 ms	$T_{target}$ = 0.532 ms

**Table 3:** Configurations of the LCDP ablation experiments.

Experiment ID	Model Configuration	Output-Layer Knowledge Distillation	LCAO	LFD	DOSP	Purpose
A0	Base	√	×	×	×	Reference lightweight student model for evaluating the gains from other modules
A1	Base + LCAO	√	√	×	×	Evaluate the effect of latency-constrained optimization on the accuracy-efficiency trade-off
A2	Base + LFD	√	×	√	×	Evaluate whether local feature distillation improves the representational capacity of the lightweight model
A3	Base + DOSP	√	×	×	√	Evaluate the contribution of dynamic structured sparsification to deployment efficiency
A4	Base + LCAO + LFD	√	√	√	×	Analyze the coordination between latency constraints and feature transfer
A5	Base + LCAO + DOSP	√	√	×	√	Analyze the joint effect of latency optimization and structured sparsification
A6	Base + LFD + DOSP	√	×	√	√	Analyze the combination of feature compensation and structured sparsification
A7	Full LCDP	√	√	√	√	Evaluate the overall optimization capability of the complete LCDP framework

Note: LCAO denotes latency-constrained adaptive optimization, LFD denotes local feature distillation, and DOSP denotes deployment-oriented dynamic structural pruning. A check mark and a cross indicate that the corresponding module is enabled and disabled, respectively.

**Table 4:** Comparison of forecasting accuracy and model complexity on the ETTh1, ETTm1, and ETTm2 test sets, with inference latency measured on CPU 1 (Intel Core Ultra 5 225H).

Model	Params	MACs	ETTh1			ETTm1			ETTm2		
			Latency (ms)	MSE	MAE	Latency (ms)	MSE	MAE	Latency (ms)	MSE	MAE
LCDP	9416	50,848	0.426 ± 0.015	0.096	0.230	0.494 ± 0.027	0.046	0.152	0.524 ± 0.023	0.101	0.227
KAN	4609	442,368	0.523 ± 0.025	0.149	0.287	0.583 ± 0.019	0.066	0.191	0.715 ± 0.045	0.170	0.318
Token Merging	15,592	108,857	17.459 ± 1.233	0.092	0.227	17.285 ± 0.072	0.048	0.159	17.580 ± 0.061	0.102	0.230
DLinear	18,632	130,368	1.044 ± 0.082	0.113	0.249	0.241 ± 0.008	0.047	0.154	1.016 ± 0.042	0.105	0.226
BESN	301,596	24,384,000	11.034 ± 0.770	0.140	0.284	11.078 ± 0.369	0.075	0.204	8.586 ± 0.370	0.231	0.373
GRU	64,992	4,988,928	8.616 ± 0.566	0.098	0.235	6.854 ± 0.213	0.047	0.155	8.608 ± 0.447	0.109	0.246
Transformer	696,544	12,429,312	1.837 ± 0.106	0.139	0.292	1.673 ± 0.018	0.069	0.207	1.697 ± 0.024	0.105	0.244
Informer	11,325,953	1,092,593,664	33.037 ± 2.185	0.168	0.320	33.195 ± 0.063	0.099	0.246	33.485 ± 0.060	0.111	0.249

**Table 5:** Comparison of forecasting accuracy and model complexity on the ETTh1, ETTm1, and ETTm2 test sets, with inference latency measured on CPU 2 (Intel Xeon Gold 6133 @ 2.50 GHz).

Model	Params	MACs	ETTh1			ETTm1			ETTm2		
			Latency (ms)	MSE	MAE	Latency (ms)	MSE	MAE	Latency (ms)	MSE	MAE
LCDP	9416	50,848	0.128 ± 0.003	0.094	0.230	0.130 ± 0.001	0.046	0.152	0.129 ± 0.001	0.101	0.227
KAN	4609	442,368	0.159 ± 0.001	0.149	0.287	0.162 ± 0.004	0.066	0.191	0.158 ± 0.003	0.170	0.318
Token Merging	15,592	108,857	0.997 ± 0.002	0.092	0.228	1.009 ± 0.016	0.048	0.159	1.172 ± 0.007	0.102	0.230
DLinear	18,632	130,368	0.418 ± 0.002	0.113	0.249	0.435 ± 0.012	0.047	0.154	0.534 ± 0.005	0.105	0.226
BESN	301,596	24,384,000	10.975 ± 0.085	0.140	0.284	10.369 ± 0.160	0.075	0.204	9.437 ± 0.106	0.231	0.373
GRU	64,992	4,988,928	4.675 ± 0.018	0.098	0.235	4.847 ± 0.107	0.047	0.155	4.536 ± 0.012	0.109	0.246
Transformer	696,544	12,429,312	1.478 ± 0.009	0.139	0.292	1.561 ± 0.033	0.069	0.207	1.387 ± 0.003	0.105	0.244
Informer	11,325,953	1,092,593,664	20.149 ± 0.488	0.169	0.321	19.950 ± 0.199	0.099	0.246	19.157 ± 0.503	0.111	0.249

**Table 6:** Ablation results for the individual LCDP modules and the complete framework on the ETTh1, ETTm1, and ETTm2 test sets.

ID	Model	ETTh1			ETTm1			ETTm2		
		Latency (ms)	MSE	MAE	Latency (ms)	MSE	MAE	Latency (ms)	MSE	MAE
A0	Base	0.448 ± 0.028	0.09709	0.23755	0.560 ± 0.048	0.04560	0.15156	0.548 ± 0.015	0.10080	0.22711
A1	Base + LCAO	0.482 ± 0.011	0.10226	0.24443	0.556 ± 0.014	0.04560	0.15156	0.540 ± 0.039	0.10080	0.22711
A2	Base + LFD	0.541 ± 0.039	0.10291	0.24601	0.549 ± 0.037	0.04558	0.15184	0.549 ± 0.029	0.10084	0.22688
A3	Base + DOSP	0.479 ± 0.026	0.10230	0.24392	0.529 ± 0.030	0.04549	0.15135	0.546 ± 0.044	0.10083	0.22692
A7	Full LCDP	0.426 ± 0.015	0.09571	0.23047	0.494 ± 0.027	0.04591	0.15220	0.524 ± 0.023	0.10083	0.22685

**Table 7:** Ablation results for combinations of LCDP modules and the complete framework on the ETTh1, ETTm1, and ETTm2 test sets.

ID	Model	ETTh1			ETTm1			ETTm2		
		Latency (ms)	MSE	MAE	Latency (ms)	MSE	MAE	Latency (ms)	MSE	MAE
A4	Base + LCAO + LFD	0.477 ± 0.011	0.09489	0.23099	0.536 ± 0.015	0.04558	0.15184	0.549 ± 0.023	0.10084	0.22688
A5	Base + LCAO + DOSP	0.468 ± 0.020	0.10586	0.24890	0.566 ± 0.011	0.04549	0.15135	0.555 ± 0.019	0.10083	0.22692
A6	Base + LFD + DOSP	0.469 ± 0.037	0.09470	0.23122	0.525 ± 0.021	0.04548	0.15160	0.525 ± 0.009	0.10083	0.22685
A7	Full LCDP	0.426 ± 0.015	0.09571	0.23047	0.494 ± 0.027	0.04591	0.15220	0.524 ± 0.023	0.10083	0.22685

## Data Availability

The data that support the findings of this study are available from the corresponding author upon reasonable request.
